# Psychosocial characteristics of the general population who habitually use hypnotics: Results from a national survey on drug use among the Japanese

**DOI:** 10.1002/pcn5.208

**Published:** 2024-07-09

**Authors:** Satomi Mizuno, Takuya Shimane, Satoshi Inoura, Toshihiko Matsumoto

**Affiliations:** ^1^ Department of Drug Dependence Research National Institute of Mental Health Kodaira Japan; ^2^ Department of Forensic Medicine, Graduate School of Medicine The University of Tokyo Tokyo Japan

**Keywords:** anti‐anxiety agents, illegal drugs, painkillers, sleep disorders, smoking

## Abstract

**Aim:**

The aim of this study was to examine the characteristics of habitual hypnotic users in Japan.

**Methods:**

This nationwide, cross‐sectional survey used self‐administered questionnaires. Data were collected from four national surveys conducted every 2 years between 2015 and 2021. The participants were Japanese individuals who had taken prescription hypnotics in the past year or had never taken them. We divided 13,396 participants into three groups to compare the social background and status of taking medication and controlled drugs, drinking, and smoking among the three groups: people who use hypnotics habitually daily (habitual hypnotic users [HUs]), people who use them only occasionally (occasional hypnotic users [OUs]), and people who do not use them (hypnotic non‐users [NUs]). We compared the perception of using hypnotics between the HU and OU groups.

**Results:**

HUs were more likely to be older, unemployed, and to habitually use anxiolytics and analgesics than NUs. The main reasons for taking anxiolytics in HUs were alleviating insomnia and reducing anxiety, whereas the main reason for taking analgesics was improving joint pain. Additionally, the HU group had a higher proportion of habitual smokers than the OU group. There was no difference in drinking status or taking of controlled drugs among the three groups. HUs were more likely to use hypnotics and to have concerns about their side‐effects than OUs.

**Conclusion:**

HUs were more likely to be unemployed, habitually use anxiolytics and analgesics, smoke heavily, and take hypnotic drugs with concerns regarding side‐effects. These results may help encourage the appropriate use of hypnotics.

## INTRODUCTION

Pharmacotherapy with hypnotics is one of the most common treatments for insomnia. However, some hypnotic drugs, depending on their type, have been reported to cause health problems, such as reduced motor and cognitive functions and mood disorders, when used in large doses and on a long‐term regular basis.^1^, ^2^, ^3^, ^4^, ^5^, ^6^, ^7^, ^8^ In Japan, benzodiazepine receptor agonists, which cause use disorders, are prescribed more frequently than in other countries, which is a problem.^9^, ^10^, ^11^ In response to this clinical situation, various measures for the appropriate prescription of hypnotics have been considered, such as medical fee revisions for the polypharmacy of hypnotics.^12^, ^13^, ^14^, ^15^, ^16^, ^17^, ^18^ In particular, information on the characteristics of habitual hypnotics users (HUs) is crucial to developing guidelines to achieve both efficient treatment of insomnia and prevention of health problems caused by hypnotics.

In previous studies conducted in Japan,^12^, ^13^, ^14^, ^15^, ^16^, ^17^, ^18^ the prescription status of patients prescribed hypnotics by medical institutions was investigated using medical record information on hypnotic prescriptions. The characteristics of hypnotic users were identified from the prescription status of hypnotics by component, the department in which hypnotics were prescribed, and the duration of the prescription. The mean age of the patients for whom hypnotics were prescribed was approximately 40 years, with benzodiazepine receptor agonists being the most commonly prescribed drugs; the average duration of prescription of hypnotics was approximately 3 months. The proportion of patients for whom hypnotics were prescribed for more than 12 consecutive months was approximately 10%.^16^, ^17^, ^18^ Previous studies have reported that patients with higher prescribed doses of hypnotics at the time of initial prescription are characterized by prolonged hypnotic use.^9^, ^18^, ^19^

Previous studies investigating hypnotic prescription status based on medical information had excellent study designs because of the large sample sizes.^9^, ^12^, ^13^, ^15^, ^17^, ^18^ They helped in understanding the characteristics of patients prescribed hypnotics and the status of prescriptions in clinics and hospitals. However, given that information in the previous studies relied solely on medical claims data, the results may not accurately reflect individual drug‐taking behaviors. Previous studies have not provided sufficient information on the patients' backgrounds. In particular, little is known about the status of consumption of other substances by HUs, despite the anticipated association between the characteristics of HUs and insomnia patients because of the presence of psychiatric disorders,^20^, ^21^, ^22^, ^23^, ^24^, ^25^, ^26^, ^27^ chronic pain,^22^, ^28^, ^29^, ^30^, ^31^ heavy drinking,^1^, ^32^ smoking,^1^, ^33^, ^34^, ^35^ and use of controlled drugs,^32^ such as cannabis, cocaine, and opiates, which have been reported as factors that can cause insomnia symptoms. Understanding the psychosocial characteristics of the general population who habitually consume hypnotics could be helpful in policymaking regarding the appropriate use of hypnotics.

We conducted this study using data from a national epidemiological study using a self‐administered questionnaire. The aim was to explore the characteristics of HUs by comparing HUs with occasional users (OUs) and non‐users (NUs).

## METHODS

### Design

This study was a nationwide cross‐sectional study using self‐administered questionnaires to examine the characteristics of HUs in Japan. Data were collected from four national surveys conducted every 2 years between 2015 and 2021,^36^, ^37^, ^38^, ^39^ called the Nationwide General Population Surveys. We divided participants into three groups of individuals who use hypnotics habitually daily (HUs), those who use them only occasionally (OUs), and those who do not use them (NUs) to compare the social background and medication status.

### Description of the nationwide general population survey on drug use in Japan

The Nationwide General Population Survey on Drug Use in Japan has been conducted every 2 years since 1995 among all citizens aged between 15 and 64 years to investigate the trend and prevalence of illicit drugs and medicines, including hypnotics usage, alcohol consumption, and smoking in Japan. The results obtained from the survey are used as a basis for developing policies against drug abuse, such as the Fifth Five‐Year Drug Abuse Prevention Strategy, which is at the heart of the fight against drug abuse in Japan.

The survey methodology has been standardized as in the epidemiological surveys of other countries, such as the National Survey on Drug Use and Health in the United States, the European Monitoring Centre for Drugs and Drug Addiction in Europe, and the Canadian Tobacco and Alcohol and Drugs Survey in Canada. Previous reports have described the procedure in detail.^36^, ^37^, ^38^, ^39^ Briefly, the survey methodology was as follows: participants were selected from nationwide residents by stratified two‐stage random sampling at first. Survey descriptions and questionnaires were sent to the participants in advance. Only consenting participants completed a self‐administered questionnaire on the survey items. No personal information was collected because this study was a self‐administered anonymous survey. An external research company selected the participants, and the authors only collected non‐linkable anonymized information of participants.

### Variables

Variables evaluated in the Nationwide General Population Surveys included age; sex; residential location; educational level; unemployment; where hypnotics were obtained; the frequency of hypnotic use; the perception toward and the reason for taking medication, including hypnotics, anxiolytics, analgesics, alcohol, and tobacco; and experience with controlled drugs.

Residential location was classified into three categories: cities with a population of ≥1 million (metropolitan areas), between 200,000 and 1 million (regional urban areas), and ≤200,000 (underpopulated areas); location was also categorized based on differences in the number of hospitals and clinics. We evaluated whether participants had completed compulsory education and whether they were unemployed. We defined unemployed individuals as those who currently did not have a job to earn income at all, excluding students and those who are full‐time, part‐time, or self‐employed. Because economic status and physical conditions vary among generations, we chose these variables based on previous studies on patients with insomnia and patients prescribed hypnotics.^40^, ^41^, ^42^, ^43^

Data on the frequency of hypnotic drug use were obtained from the participants via questionnaires including the following eight items: (1) never, (2) several times a year, (3) about once every 2 months, (4) once or twice a month, (5) several times a month, (6) once or twice a week, (7) three to six times a week, and (8) every day over the past year. Based on the eight categories of hypnotic use frequency, we next classified the participants into three groups. Almost daily habitual hypnotic users (as previously defined, HUs) were people taking hypnotics more than 3 days per week (Categories 7 and 8); occasional hypnotic users (as previously defined, OUs) were people taking hypnotics less than 3 days per week (Categories 2–6); and non‐users (as previously defined, NUs) were people who never take hypnotics (Category 1). People who continued to use hypnotics 3 or more days a week in the past year were considered HUs because they were assumed to be the same as previously reported “patients who have been regularly prescribed hypnotics for a long period.”^9^ Additionally, we investigated perceptions regarding side‐effects of hypnotics. We used three categories to examine participants' perceptions about the use of hypnotics when they needed hypnotics for insomnia: (1) no worry about side‐effects, (2) worry about the side‐effects, and (3) limiting use due to worry. Some hypnotics prescribed in Japan, such as benzodiazepines, cause severe dependence and side‐effects in habitual users.^44^

We assessed the frequency of anxiolytic and analgesic use in the past year. We investigated whether participants habitually took anxiolytics or analgesics more than 3 days a week in the past year and the reasons for taking them. We classified the reasons for the use of anxiolytics into three categories: to alleviate insomnia; as mood stabilizers to reduce anxiety or stress; and for nontherapeutic purposes, such as recreation and abuse. The reasons for using analgesics were classified into four categories: to alleviate headaches; to alleviate joint pain; to alleviate pain other than headaches and joint pain, such as menstrual pain and tooth pain; and for nontherapeutic purposes, such as recreation and abuse.

We assessed the frequency of drinking alcohol and smoking in the past 30 days. We investigated whether participants had been habitually drinking more than 3 days a week and whether participants had been habitually smoking more than 20 days a month, based on the National Health and Nutrition Survey of the Ministry of Health, Labour and Welfare.^43^ Furthermore, we assessed the use of controlled drugs, such as marijuana, methamphetamine, inhalants, 3,4‐methylenedioxymethamphetamine, cocaine, heroin, new psychotropic substances, and lysergic acid diethylamide in Japan in the past year.

### Inclusion and exclusion criteria

From among the total participants in the Nationwide General Population Surveys 2015–2021, we selected participants who provided consent and completed all questions. Given that this study focused on participants using prescribed hypnotics for insomnia treatment, we excluded people who had obtained hypnotics from sources other than hospitals and clinics, such as drugstores, the internet, family members, or acquaintances, based on the information regarding where medications were obtained. Similarly, because we aimed to assess the status of prescribed anxiolytics, we excluded people who had obtained anxiolytics from sources other than hospitals and clinics.

### Statistical analyses

To identify the characteristics of the HU group, we compared the HU, OU, and NU groups using analysis of variance for continuous variables and the chi‐squared or Fisher's exact test for categorical variables. Furthermore, we used the chi‐squared or Fisher's exact test for categorical variables associated with perceptions about using hypnotics to compare the HU and OU groups. All statistical analyses were performed using R Version 4.2.3, statistical software (R Foundation for Statistical Computing). With Bonferroni correction, *p* < 0.016 was considered to indicate statistical significance. The sample size of this study was large. Effect sizes were calculated.

In each nationwide general survey, we calculated the estimated proportion of people who used medications.^36^, ^37^, ^38^, ^39^ As a reference to ensure that there is no bias in the proportion of regular hypnotic drug users in each survey year, the estimated proportion of HUs was recorded. The point and interval estimates (95% confidence intervals) were calculated using IBM SPSS Complex Samples (IBM Corp.) for the estimated number of people who habitually used hypnotics. Details of the analysis methodology and results can be found in previous reports.^36^, ^37^, ^38^, ^39^

### Ethical issues

The Nationwide General Population Survey on Drug Use and the secondary analysis study using the surveys were approved by the ethics committee of our institution (A2023‐031). In the Nationwide General Population Surveys, informed consent was not obtained from participants as an opt‐out approach was adopted. By compiling the necessary information about the conduct of the survey in a public notice document approved by the Ethics Committee and posting it on the public website of our research facility, we guaranteed the participants the right to refuse to participate in the survey. This also ensured that participants could withdraw from the survey, even after submitting the questionnaire upon request. The method for withdrawing research cooperation was described in the public notice document, survey descriptions, and the questionnaire form. The documents included the following information: “As responses to the questionnaire form are anonymous, individuals will not be identified by questionnaire,” “Responses to the questionnaire form will be voluntary,” and “There will be no disadvantages for not cooperating in the research.” The participants' consent to participate in the survey was confirmed by their completion of the questionnaire form or using the online consent confirmation box. This study was conducted according to the tenets of the Declaration of Helsinki.

## RESULTS

**Figure 1:**
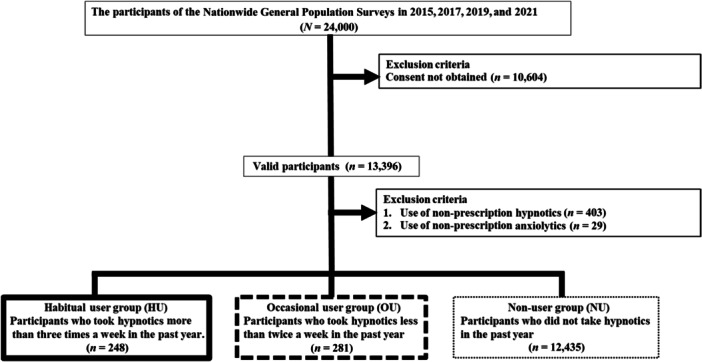
Flowchart of participant selection.

From among the 24,000 participants in the Nationwide General Population Surveys 2015–2021, we excluded 10,604 who did not provide consent or complete all questions, 403 who had obtained nonprescription hypnotics, and 29 who had obtained nonprescription anxiolytics. We selected 13,396 participants who were then divided into three groups, based on eight categories according to the frequency of hypnotics use: 12,435, 151, 24, 32, 38, 36, 44, and 204 participants in Categories 1–8, respectively. The numbers of participants in each group were: HUs, 248 (Categories 7 and 8); OUs, 281 (Categories 2–6); and NUs, 12,435 (Category 1) (Figure 1).

Tables 1 and 2 present the multiple comparison test results for the three groups for each variable. The variables that were significantly different in the HU group compared to the OU or NU groups were age, unemployment, habitual use of anxiolytics and analgesics, the reason for taking anxiolytics and analgesics, and smoking (Table 1). No significant differences in the proportion of habitual drinkers and people using controlled drugs among the three groups were observed.

**Table 1:** Comparison of characteristics between the habitual user, occasional user, and non‐user groups.

	HUs	OUs	NUs		
	*n* = 248	*n* = 281	*n* = 12,435	*p*‐value	Effect size
Women	148 (59.7)	188 (66.9)	6,404 (51.5)	<0.001	0.050
Age (Mean (SD), years)	48.2 (11.5)	47.5 (12.3)	43.2 (13.8)	<0.001	0.005
Living location				0.530	0.011
Metropolitan	73 (29.4)	83 (29.5)	3293 (26.5)		
Regional urban	54 (21.8)	67 (23.8)	3124 (25.1)		
Underpopulated area	121 (48.8)	131 (46.6)	6018 (48.4)		
Unemployment	55 (22.3)	28 (10.0)	533 (4.3)	<0.001	0.121
Completed compulsory education	237 (95.6)	263 (93.6)	11,298 (90.9)	0.011	0.026
Habitually taking anxiolytics	144 (59.0)	55 (19.8)	141 (1.1)	<0.001	0.517
Reason for taking anxiolytics					
Insomnia	117 (47.6)	68 (24.2)	71 (0.6)	<0.001	0.518
Anxiety	116 (47.2)	82 (29.2)	181 (1.5)	<0.001	0.437
Non‐therapeutic purposes	0 (0.0)	0 (0.0)	1 (0.0)	0.979	0.002
Habitually taking analgesics	35 (14.1)	21 (7.5)	282 (2.3)	<0.001	0.112
Reason for taking analgesics					
Headache	120 (48.4)	149 (53.2)	5,017 (40.4)	<0.001	0.044
Arthritis	86 (34.7)	92 (32.9)	2275 (18.3)	<0.001	0.078
Pain other than headache and joint pain	65 (26.2)	68 (24.3)	2831 (22.8)	0.381	0.012
Non‐therapeutic purposes	0 (0.0)	0 (0.0)	7 (0.1)	0.862	0.005
Habitual smoking	63 (25.6)	37 (13.3)	2446 (19.8)	0.002	0.031
Habitual drinking	52 (21.1)	53 (18.9)	2771 (22.3)	0.357	0.013
Using controlled drugs	1 (0.4)	0 (0.0)	33 (0.3)	0.628	0.008

*Note*: Metropolitan: ≥1 million; regional urban: between 200,000 and 1 million; underpopulated area: ≤200,000.

Habitual anxiolytic use: frequency of taking anxiolytics >3 times every week. Habitual consumption of analgesics: frequency of taking analgesics >3 times per week. Habitual drinking: frequency of drinking >3 times per week. Habitual smoking: smoking frequency >20 days/month. *p* < 0.05 was considered to indicate statistical significance.

Abbreviations: 95% CI, 95% confidence interval; HUs, habitual users of hypnotics; NUs, non‐users of hypnotics; OUs, occasional users of hypnotics; SD, standard deviation.

**Table 2:** Multiple comparison test results of the OUs and NUs against the HUs for sex, age, unemployment, status of taking anxiolytics and analgesics, and smoking

	OUs		NUs	
	*p*‐value	Effect size	*p*‐value	Effect size
Women	0.308	0.075	0.039	0.023
Age	1.000	0.001	<0.001	0.002
Unemployment	<0.001	0.168	<0.001	0.118
Completed compulsory education	1.000	0.043	0.043	0.023
Habitually taking anxiolytics	<0.001	0.403	<0.001	0.536
Reason for taking anxiolytics				
Insomnia	<0.001	0.244	<0.001	0.185
Anxiety	<0.001	0.536	<0.001	0.417
Habitually taking analgesics	more	0.107	<0.001	0.105
Reason for taking analgesics				
Headache	0.920	0.048	0.040	0.023
Arthritis	1.000	0.019	<0.001	0.058
Habitual smoking	0.001	0.157	0.091	0.020

*Note*: Habitual use of anxiolytics: frequency of use of anxiolytics >3 times a week in the past year. Habitual use of analgesics: frequency of use of analgesics >3 times a week in the past year. Habitual smoking: smoking frequency >20 days/month. Statistical significance was set at *p* < 0.0167.

Abbreviations: HUs, habitual users of hypnotics; NUs, non‐users; OUs, occasional users of hypnotics.

HUs were more likely to be female than NUs (59.7% vs 51.5%, *p* = 0.039); however, the difference was not significant after Bonferroni correction. HUs were significantly older (48.23 years [standard deviation: 11.50] vs. 43.22 [13.76], *p* < 0.001), and they had a higher proportion of those who were unemployed (22.3% vs 4.3%, *p* < 0.001), and those who habitually used anxiolytics (59.0% vs 1.1%, *p* < 0.001) and analgesics (14.1% vs 2.3%, *p* < 0.001) than NUs. Additionally, compared with NUs, a higher proportion of HUs took anxiolytics for insomnia (47.6% vs 0.6%, *p* < 0.001) and anxiety (47.2% vs 1.5%, *p* < 0.001) and analgesics for joint pain (37.4% vs 18.3%, *p* < 0.001) **(**Table 2 **)**.

HUs were significantly more likely to be unemployed (HUs > OUs: 22.3% > 10.0%, *p* < 0.001) and habitually use anxiolytics (59.0% > 19.8%, *p* < 0.001) and analgesics (14.1% > 7.5%, *p* = 0.049) than OUs, similar to the results for NUs. HUs were more likely to take anxiolytics for insomnia (47.6% vs 24.2%, *p* < 0.001) and anxiety (47.2% vs 29.2%, *p* < 0.001) than OUs. HUs included more habitual smokers (25.6% vs 13.3%, *p* = 0.001) than OUs.

HUs were more likely to use hypnotics despite concerns about their side‐effects (HUs > OUs: 43.5% > 32.0%, *p* = 0.008). There was no significant difference between the HU and OU groups in the proportion of people who used hypnotics without concerns about side‐effects (Table 3).

**Table 3:** Perception about using hypnotics between the HU and OU groups when they needed hypnotics for insomnia

	HUs	OUs		
	*n* = 248	*n* = 281	*p*‐value	Effect size
Using without worrying about the side‐effects	135 (54.4)	131 (46.6)	0.088	0.078
Using while worrying about the side‐effects	108 (43.5)	90 (32.0)	0.008	0.119
Using as little as possible because of worrying about the side‐effects	5 (2.0)	60 (21.4)	<0.001	0.232

*Note*: Data are presented as *n* (%) unless otherwise indicated.

We considered *p* < 0.05 to indicate statistical significance.

Abbreviations: HUs, habitual users of hypnotics; OUs, occasional users of hypnotics.

The estimated proportion of HUs in Japan was approximately 2%, equal between the survey years of 2015, 2017, 2019, and 2021.^36^, ^37^, ^38^, ^39^ The number of participants for each survey year is shown in Figure S1.

## DISCUSSION

To the best of our knowledge, this study, using the Nationwide General Population Surveys, is the first to show the psychosocial characteristics of the general population who are HUs compared with those of OUs and NUs.

### Background: age, women, unemployment

This study showed that HUs were older and had a higher proportion of people unemployed than NUs. Our results are similar to previous observations of patients with chronic insomnia,^23^, ^34^, ^40^ apart from no significant differences in the proportion of women. The finding that there were no sex differences between HUs and OUs supports the findings of previous Japanese studies using medical information.^23^, ^34^, ^40^ Although the age ranges differed between this study and previous research, the previous studies similarly showed no sex differences between patients who were prescribed long‐term hypnotics and those who were not.^17^, ^18^

In this study, HUs had a higher proportion of unemployed people than OUs. Our results showed that the frequency of hypnotic use was associated with employment status, suggesting that HUs may face limitations in performing their jobs because they cannot sleep without using hypnotic drugs almost every day. Another potential explanation for the increased unemployment is that HUs might have more severe insomnia symptoms than OUs and may have more comorbid psychiatric disorders, such as depression,^41^ which can also affect employment.

### Anxiolytics

Among the three groups, the proportion of people who habitually took anxiolytics daily over 1 year was higher among HUs. Previous studies have also reported that patients who were prescribed long‐term hypnotics tended to be prescribed anxiolytics,^13^, ^14^, ^15^ consistent with our results. Previous studies have reported that insomnia increases stress, and insomnia symptoms coexist with depression and other psychiatric disorders.^8^, ^22^, ^25^, ^34^, ^45^ Furthermore, according to Japanese receipt data, prescriptions combining anxiolytics and hypnotics are commonly prescribed in specialized outpatient clinics, such as those specializing in psychosomatic medicine and psychiatry.^14^, ^15^ In Japan, combinations of antidepressants, anxiolytics, and hypnotics are often prescribed to patients with treatment‐resistant insomnia who do not respond to hypnotics alone and require long‐term use. These reasons, based on the current clinical setting, might have resulted in the significant association observed in the present study between the use of antidepressants and the long‐term use of hypnotics.

### Analgesics

HUs used analgesics more frequently than OUs. The main reason for using analgesics was to relieve headaches and joint pain, and the reasons did not differ between HUs and OUs. HUs used analgesics more habitually than OUs, despite no difference in the reasons for using analgesics between HUs and OUs, suggesting that HUs may have chronic pain. Previous studies have suggested an interrelationship between sleep disturbance and chronic pain.^28^, ^29^, ^31^ Sleep disturbances can lead to detrimental psychological and behavioral effects, such as increased depressive and anxiety symptoms, which exacerbate pain. Conversely, increased pain stimulates the sympathetic nervous system and increases the inflammatory response, causing sleep disturbances.^29^ Regardless of the reason for our results, healthcare professionals should pay attention to the pain status of patients who consume hypnotics. Working with psychologists and pain clinic specialists may also be helpful. Some cases have been reported in which insomnia was resolved through cognitive behavioral therapy and chronic pain control in collaboration with these professionals.^29^, ^30^, ^31^, ^46^, ^47^

### Smoking

HUs were more likely to be habitual smokers than OUs. Smoking reduces cardiopulmonary function and is associated with sleep apnea and lung‐related diseases.^23^, ^35^ Previous studies have reported that chronic insomnia is associated with upper airway inflammatory diseases, such as asthma and laryngopharyngitis.^33^ The side‐effects of smoking could have caused sleep disturbances, which might have led to habitual use of hypnotics. Another potential reason for this result is that the HUs in this study may have had a higher proportion of patients with psychological disorders. Previous studies reported a higher proportion of habitual smokers among patients with psychological disorders.^48^, ^49^ Patients with mental disorders might believe that smoking is necessary for self‐medication because nicotine is a powerful reinforcer that temporarily increases concentration and alertness and reduces stress, anxiety, and other symptoms when taken into the body.^48^ The latest guidelines mention the relationship between smoking and sleep disturbances as well as alcohol consumption.^50^, ^51^, ^52^ Considering these results, the guidelines should be followed more aggressively than in the past, and patients prescribed hypnotic medications should be educated about the side‐effects of smoking on sleep and advised to quit smoking if necessary.

### Drinking

The proportion of habitual drinkers did not differ between the HUs, OUs, and NUs. Our results suggest that HUs in Japan could be as adherent as NUs regarding prescription hypnotics while following the advice of doctors. Although alcohol consumption has a sleep‐inducing effect, guidelines on insomnia have recommended against alcohol consumption because its diuretic effect is known to cause mid‐onset and early awakening, leading to sleep disturbances.^1^, ^32^ Popular benzodiazepines and benzodiazepine‐like agents are often avoided in individuals with alcohol use disorders because of their addictive potential and increased risk of toxicity or overdose when these medications are mixed with alcohol.^1^, ^32^

### Controlled drugs

The proportion of people using controlled drugs did not differ among HUs, OUs, and NUs. HUs may comply with advice about controlled drug use because their use of other controlled drugs, such as cannabis, cocaine, and opioids, can cause sleep disturbances, similar to drinking alcohol.^32^ Additionally, Japan has a setting in which the crackdown on controlled drugs is rigorous. The proportion of people who have used controlled drugs in the past year is as low as 0.3%,^36^, ^37^, ^38^, ^39^ which might also be the reason for the lack of significant differences among the three groups. There may also be a reporting bias due to the strong stigma against controlled substance users.

### Perception of using hypnotics between HUs and OUs

There was no significant difference between users in the proportion of people who used hypnotics without worrying about their side‐effects. HUs were more likely to use hypnotics while worrying about side‐effects than OUs. The results of this study are similar to those of a previous study of 104 patients taking benzodiazepine receptor agonists, such as hypnotics and anxiolytics.^44^ This study had the largest sample size of previous awareness surveys of patients conducted in Japan. It might support the consensus about HUs because the data were obtained through national surveys.

Patients' perceptions toward hypnotics for insomnia may influence the habitual use of hypnotics as well as the physician's perception toward hypnotics for insomnia and the physician's attitude toward discontinuing hypnotics. One reason for the attitude of patients who are concerned about the side‐effects of hypnotic drug use but still use hypnotic drugs habitually may be that HUs feel that escaping the distress associated with not sleeping is more important than the fear of future side‐effects of hypnotics. Given our finding that patients took hypnotics, despite their fear of side‐effects, when considering treatment for insomnia or prescription of hypnotics, doctors should also consider patients' perceptions of hypnotics. Even if patients continue to use hypnotics, physicians might reduce potential harm by offering alternative treatment. For example, it might be more appropriate to offer patients cognitive behavioral therapy to improve insomnia or to switch to hypnotics with fewer side‐effects to better suit their needs,^50^ as already mentioned in the guidelines,^50^ rather than strictly forcing these patients to stop taking illicit hypnotics.

### Limitations

This study has some limitations. First, this study did not investigate the use of hypnotics according to the components of each hypnotic because we used a self‐administered questionnaire. Despite the attention paid to the harm caused by long‐term regular use of benzodiazepines, this study could not investigate the characteristics of habitual users of benzodiazepines specifically. However, because most hypnotics prescribed in Japan are benzodiazepines, the study participants likely included many benzodiazepine users. This study may be helpful in indirectly understanding the characteristics of habitual users of benzodiazepine receptor agonists. Second, we investigated only known or reported insomnia‐related factors; other unknown factors may be associated with habitual hypnotic use. However, this study investigated more variables simultaneously than previous studies did, including the frequency and reasons for taking medication. Finally, data were collected by self‐reporting, which might have led to a reporting bias.

## CONCLUSION

Herein, we described the characteristics of HUs in Japan. HUs were older than NUs. HUs had the characteristics of being unemployed and habitual users of anxiolytics and analgesics, smoking heavily, and using hypnotics despite worrying about their side‐effects, in contrast to OUs. These findings could be used to inform the appropriate use of hypnotics.

## AUTHOR CONTRIBUTIONS

Takuya Shimane and Satomi Mizuno designed preliminary experiments. Takuya Shimane, Satoshi Inoura, and Satomi Mizuno recruited the participants and collected the data. Takuya Shimane, Satoshi Inoura, and Satomi Mizuno established a database of research participants. Toshihiko Matsumoto obtained funding. Satomi Mizuno designed the study, performed statistical analyses, and wrote the manuscript. Takuya Shimane, Satoshi Inoura, and Toshihiko Matsumoto supervised the study design and manuscript writing. Satomi Mizuno wrote the initial draft of the manuscript. All authors revised and contributed to the final version of the manuscript. All authors have read and approved the final manuscript for publication.

## CONFLICT OF INTEREST STATEMENT

The authors declare no conflict of interest.

## ETHICS APPROVAL STATEMENT

The study protocol was reviewed and approved by the Ethics Committee of the National Center of Neurology and Psychiatry (NCNP) in Japan (A2023‐031). Our study conforms to the provisions of the Declaration of Helsinki.

## PATIENT CONSENT STATEMENT

In the survey, informed consent was not obtained from participants as an opt‐out approach was adopted. Even if they were in the middle of the study, the opportunity for subjects to withdraw from participating in the research was guaranteed by posting the information on how to cancel their participation in the study on the website of the Ethics Committee of the institution concerned.

## CLINICAL TRIAL REGISTRATION

N/A

## Supporting information

Supporting information.

## Data Availability

In order to protect the confidentiality of study participants, the data are unavailable.
