# Supplementary figures and images for: Psychosocial characteristics of the general population who habitually use hypnotics: Results from a national survey on drug use among the Japanese
Source: PCN Rep. 2024 Jul 9;3(3):e208. doi: 10.1002/pcn5.208 (PMC11233408; doi:10.1002/pcn5.208)

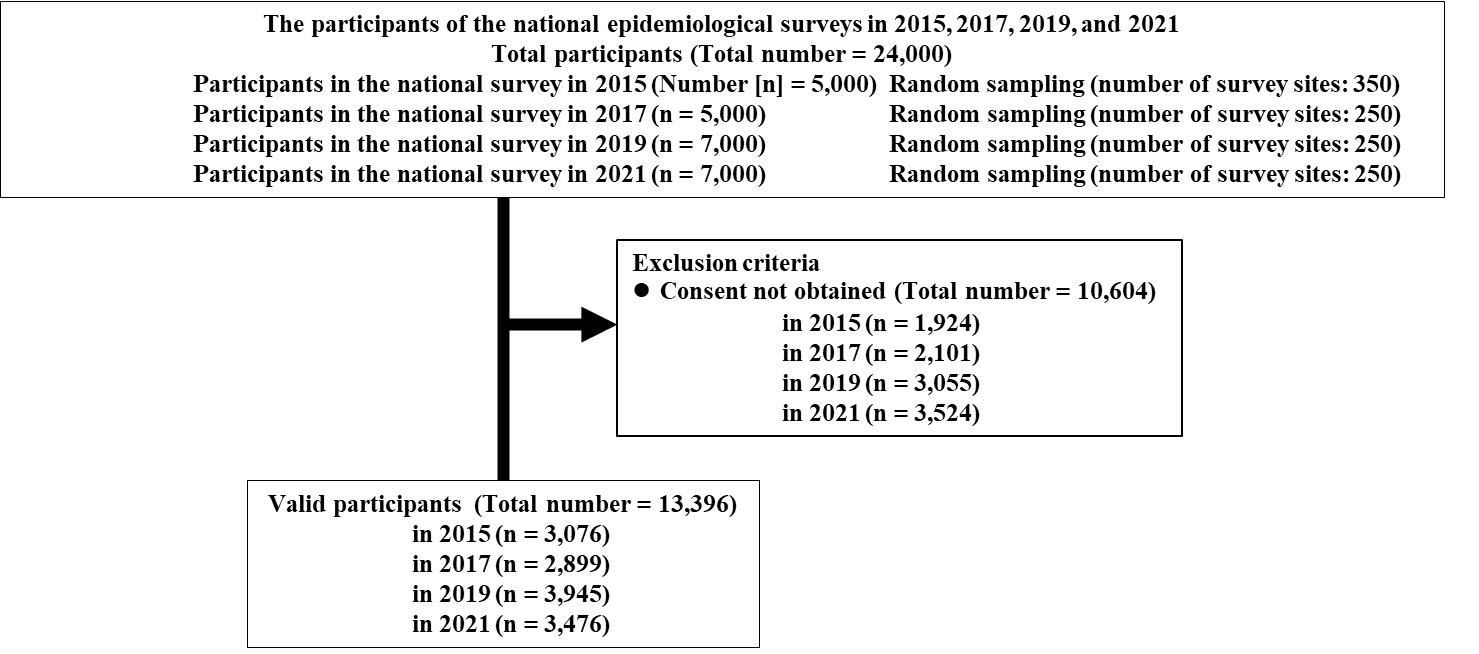
**Supporting information**

**Supplementary Figure 1**

Supplement: Supplementary file 1 — Supporting information. [file PCN5-3-e208-s001.docx]
